# Chemical Profile of *Ocotea delicata* (Lauraceae) Using Ultra High-Performance Liquid Chromatography–High-Resolution Mass Spectrometry–Global Natural Products Social Molecular Networking Workflow

**DOI:** 10.3390/plants13060859

**Published:** 2024-03-16

**Authors:** Ananda da Silva Antonio, Gustavo Ramalho Cardoso dos Santos, Henrique Marcelo Gualberto Pereira, Valdir Florêncio da Veiga-Junior, Larissa Silveira Moreira Wiedemann

**Affiliations:** 1 Department of Chemistry, Institute of Exact Sciences, Federal University of Amazonas, Avenida Rodrigo Otávio, 6200, Coroado, Manaus 69077-000, AM, Brazil; ananda.antonio@iq.ufrj.br (A.d.S.A.); gustavo.santos@iq.ufrj.br (G.R.C.d.S.); henriquemarcelo@iq.ufrj.br (H.M.G.P.);; 2 Laboratory for the Support of Technological Development, Chemistry Institute, Federal University of Rio de Janeiro, Avenida Horácio Macedo, 1281—Polo de Química—Cidade Universitária, Ilha do Fundão, Rio de Janeiro 21941-598, RJ, Brazil; 3 Department of Chemical Engineering, Military Institute of Engineering—IME, Praça General Tiburcio 80, Urca, Rio de Janeiro 22290-270, RJ, Brazil

**Keywords:** untargeted metabolomics, chemosystematic markers, chemophenetics

## Abstract

*Ocotea*, the largest genus in the Lauraceae family, encompasses numerous species of scientific interest. However, most *Ocotea* species have only been described morphologically. This study used an untargeted metabolomics workflow with UHPLC-HRMS and GNPS-FBMN to provide the first chemical evaluation of the polar specialized metabolites of *O. delicata* leaves. Leaves from three *O. delicata* specimens were extracted using ultrasound-assisted extraction with 70% ethanol. Among the examined samples, 44 metabolites, including alkaloids and flavonoids, were identified. In contrast to other *Ocotea* species, *O. delicata* has a wider diversity of kaempferol derivatives than quercetin. The biomass of the specimens showed a significant correlation with the chemical profile. The similarity among specimens was mostly determined by the concentrations of quinic acid, kaempferol glycosides, and boldine. The evaluated specimens exhibited chemical features similar to those of species classified as New World *Ocotea*, with the coexistence of aporphine and benzylisoquinoline alkaloids.

## 1. Introduction

The Amazon rainforest flora is one of the most biodiverse on the planet [1]. However, only 10% of its endemic species are thought to have any level of description. An essential tool for utilizing flora for scientific and commercial purposes is the characterization of botanical species at various organizational levels [2]. *Ocotea* (Lauraceae) is the genus with the third highest occurrence in the Amazon rainforest biome [1], a genus with a long history of ethnobotanical use, ranging from the manufacture of furniture to the treatment of illnesses. Today, several *Ocotea* species are recognized as significant sources of bioactive compounds, including those with antifungal, antimicrobial, and antiproliferative properties [3]. A famous example is the *Ocotea duckei*, which is known for its outstanding antileishmanial activity, primarily promoted by the presence of the lignoid yangambin [3]. For example, *Ocotea* species like *Ocotea puberula* (Rich.) Ness and *Ocotea odorifera* (Vell) Rohwer have traditionally been used as anti-inflammatory agents for treating acute and chronic wounds, which their bioactivity scientifically corroborated [4,5]. In the case of *O. puberula*, its anti-inflammatory capacity was even better than the commercial preparation of silver calcium alginate dressing during pre-clinical trials [5]. Besides their significance and representativeness within the Lauraceae family [6], *Ocotea* species are rarely systematically exploited due to the challenge of accurately identifying them using conventional morphological identification keys.

Alkaloids, lignoids, and flavonoids are usually the focus of chemophenetic research in the Lauraceae family [3,7]. Given their significance in chemophenetics studies and genus exploitation, several aporphinic alkaloids and neolignans have been reported as relevant chemical markers in the identification and differentiation of *Ocotea* species. However, chemical descriptions are available for only 12% of *Ocotea* species [7]. For example, *Ocotea delicata*, an endemic species of the Amazon rainforest, has never been exploited through its phylogenetic and chemical composition. The only available description pertains to its morphological features, dating back to 2000 when the species was first identified [8]. *Ocotea delicata* morphological features resemble the species *Ocotea laxa* (Nees) Mez and *Ocotea tarapotana* (Meisner) Mez [8], both of whom do not have any other description level besides the morphological either. This highlights the considerable gaps in knowledge within even the most representative genus of the Amazon rainforest biome. Hence, this study aimed to conduct the first chemical characterization of *Ocotea delicata* Vicent, an endemic and unstudied species within this genus, using an untargeted metabolomic approach to contribute to the chemical understanding of the genus.

## 2. Results

Three different specimens of *Ocotea delicata*, found within an 80 m perimeter, were selected for evaluation, considering the similarity of their environmental condition, such as the type and elevation of the relief, and sunlight incidence at the tree top. Each specimen was assigned the identifying letters A, B, and C, and their diameter at breast height (mm) can be observed in Table 1. The leaves provided an average extraction yield of 9.40 ± 2.97%. To compare the relative composition of the three distinct specimens, the peak area of each detected metabolite was normalized from 0 to 100% considering the total area of each chromatogram (relative peak area, Table 1). The relative standard deviation (RSD) (n = 3) for each sample in both examined ionization modes was less than 2.0% for retention time and 30.0% for the relative area of each peak detected. Using GNPS, 44 metabolites, including quinic acid and its derivatives, aporphine and benzylisoquinoline alkaloids, and flavonols, were putatively identified based on the similarity score of the fragmentation spectra obtained within samples to the data presented on the GNPS library (Table 1, Figures S1 and S2).

The samples exhibited distinct chromatographic profiles in the negative ionization mode (Figure S3), with major similarities observed between samples A and B. The chromatographic profiles in the positive ionization mode maintained the same similarity pattern among samples observed in the negative mode (Figure S4). Boldine and quinic acid were the compounds with the highest relative concentrations within the samples (Table 1). The high concentration of quinic acid in all samples indicates a significant presence of phenolic compounds in *O. delicata.* The phenolic content of *Ocotea delicata* (Table 1) primarily consisted of glycosylated flavonols, such as afzelin, kaempferol 7-O-glucoside, quercetin 3,7-dirhamnoside, kaempferol 3-arabinoside, and quercitrin. Additionally, four benzylisoquinoline alkaloids (coclaurine, reticuline, reticuline N-oxide, and N-methylcoclaurine) and three aporphine alkaloids (boldine, isocorydine, and N-methylaurotetanine) were detected (Table 1).

**Figure 1:**
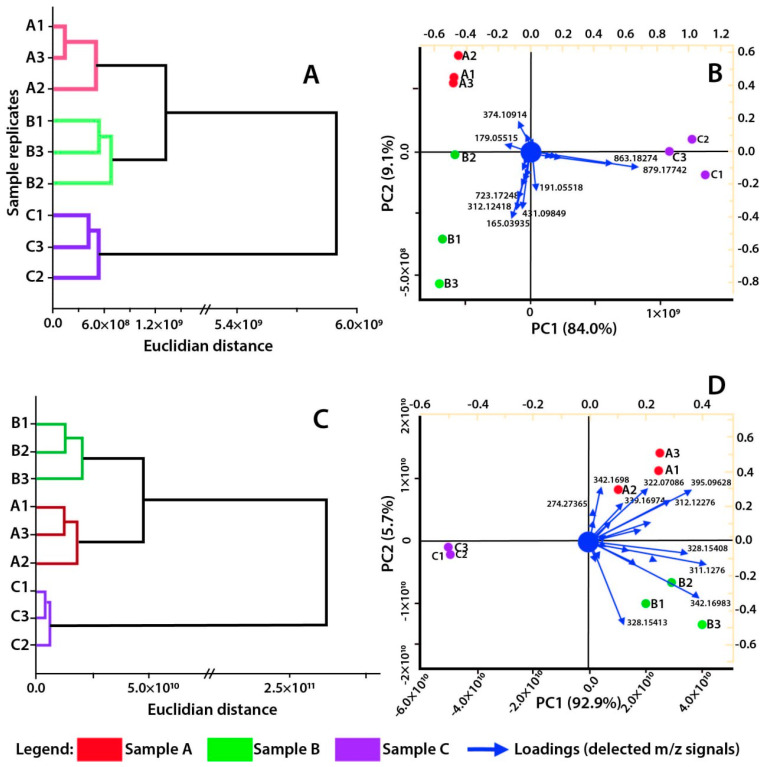
Multivariate analysis of *O. delicata* samples and replicates. (**A**) Hierarchical Clustering Analysis on negative ionization mode; (**B**) Principal Component Analysis on negative ionization mode; (**C**) Hierarchical Clustering Analysis on positive ionization mode; (**D**) Principal Component Analysis on positive ionization mode. Letters A, B, and C within each graphic represent each collected specimen, while the numbers following them are the replicates within each specimen. Loadings values in (**B**,**D**) are *m*/*z* signals detected within the samples.

A multivariate analysis was conducted with the entire chromatography dataset collected in both ionization modes to elucidate the similarity among species, as several endogenous and exogenous factors can influence the chemical composition of vegetative species [9,10]. In both ionization modes, samples A and B exhibited higher similarity to each other than to sample C (Figure 1A,B). Nine major peaks within the samples were correlated with the differentiation of the samples in the negative ionization mode (Table S2). Although these peaks could not be precisely identified, their fragmentation patterns showed m/z signals consistent with the presence of kaempferol (*m*/*z* 285, 255, and 227) and quercetin (*m*/*z* 300, 271, and 255) moieties, suggesting that they are glycosylated flavonoids. In the positive ionization mode, 11 *m*/*z* signals (Table S2) were responsible for the clustering pattern of samples (Figure 1C,D), including the reticuline (*m*/*z* 330), boldine (*m*/*z* 328), and isocorydine (*m*/*z* 342). These compounds are mainly associated with samples A and B, contributing to their similarity. It is noteworthy that these three compounds (reticuline, boldine, and isocorydine) are the major peaks within samples A and B.

## 3. Discussion

In phytochemistry, the use of time-consuming methods persists, despite advancements in unconventional extraction methods such as ultrasound-assisted extraction [11,12]. While conventional methods like Soxhlet extraction and maceration are more commonly employed, they are impractical for chemophenetics. For instance, the extraction yield achieved with the method utilized in this study was comparable to that obtained through exhaustive maceration over a 5-day period with other Lauraceae species [13], highlighting the viability of this extraction method for generating chemical fingerprints within a shorter timeframe.

Moreover, the unconventional extraction method employed in this study demonstrated reliability, as indicated by low relative standard deviation (RSD) values in the analytical responses, enabling the simultaneous extraction of a wide range of compounds in a shorter time when compared with conventional methods. It is noteworthy that, according to Begol et al. (2018) [14], RSD values within replicates should be below 20% for metabolomics studies, ensuring that the observed chemical variability arises from the inherent biological features of the samples.

*O. delicata* exhibited a phenolic content (Table 1) primarily composed of glycosylated flavonols, particularly kaempferol derivates. Despite the widespread occurrence of the *Ocotea* genus, the flavonoid profile remains poorly documented, with only 3.4% of species chemically characterized [7]. In contrast to the prevailing description of *Ocotea* flavonoids, which predominantly feature quercetin derivatives [7], *O. delicata* emerged as a notable source of kaempferol. Other *Ocotea* species containing kaempferol derivatives include *O. acutifolia*, *O. lancifolia*, *O. pulchella, O. velloziana,* and *O. caudata* [7]. It is worth noting that kaempferol and its derivatives are recognized for their therapeutic properties, including antioxidant, antitumoral, and neuroprotective effects [15].

Among the identified compounds (Table 1), alkaloids such as coclaurine, boldine, isocoroydine, and reticuline have been previously reported in other *Ocotea* species, including *O. duckeii*, *O. lancifolia*, *O. vellosiana*, *O. glaziovii,* and *O. macrophylla* [16]. While the occurrence of these alkaloids in *O. delicata* may not be particularly distinctive for differentiating *Ocotea* species, it suggests that *O. delicata* holds promise as a bioactive species. Compounds like boldine and reticuline are renowned for their biological properties, including anti-inflammatory, neuroprotective, antitumoral, and antipyretic effects [4,17].

The clustering pattern observed in the negative ionization mode (Figure 1) could be attributed to intraspecific variability among species. The assessed specimens were located within an 80 m radius of each other in a preserved native forest without abiotic stress factors. Intraspecific variability refers to chemical variations within specimens of the same species, influenced by factors such as life cycle, climate, and soil fertility [18]. No previous studies have explored intraspecific chemical variability among *Ocotea* species. Such investigations into intraspecific chemical variability have only been conducted within the Lauraceae family for the genus *Cryptocarya* [19].

The similarity observed between samples A and B could be associated with the diameter at breast height (DBH) measurements (Table 1). DBH serves as a metric for estimating growth, biomass, and the life cycle of a tree specimen [20]. Samples with higher DBH exhibited a distinct chemical composition compared to sample C (Figure 1), which, based on DBH estimation, is presumed to be the youngest specimen sampled. Two major components of sample C, salsolinol and 4-O-caffeoylquinic acid, were nearly absent in the other samples (Table 1). Conversely, sample C exhibited a minimal presence of many compounds found in samples A and B, including boldine, reticuline, and isocorydine. The prevalence of phenolic compounds may contribute to the clustering of samples A and B, suggesting that the phenolic profile could be valuable for intraspecific evaluations, despite current underutilization.

The clustering pattern observed in the positive ionization mode further supports the hypothesis that changes in the life cycle and biomass of trees can substantially influence their chemical profiles. Notably, the major metabolites in sample C (with the lowest DBH) were considerably lower than those in the largest tree samples (Table S2), as indicated by the relative peak areas of the 11 *m*/*z* signals (Figure 1D), which can be used for sample differentiation. The predominant presence of reticuline, boldine, and isocorydine in characterizing the evaluated specimens is particularly intriguing, considering that these compounds are common in the Lauraceae family [16]. Their ability to distinguish specimens of the same species suggests that they may serve as untargeted metabolomic chemical markers for further chemophenetic species differentiation and potentially for age characterization.

Although most of the identified alkaloids have been previously reported in other Lauraceae genera, including *Aniba*, *Licaria*, *Phoebe*, and *Machilus* [16], the balanced distribution of both alkaloid structural classes suggests that *O. delicata* is closely related to New World *Ocotea* species. This is because Old World *Ocotea* species exhibit a minimal expression of alkaloid structural classes other than aporphine [7].

## 4. Materials and Methods

### 4.1. Collection and Botanical Identification

Sampling was performed at a tropical non-flooded lowland forest fragment named “Sítio amostral km 37”, a primary forest preserved and maintained by the Biological Dynamics of Forest Fragments Project (PDBFF) of the National Institute of Amazonian Research (INPA) and the Smithsonian Institution. The sampling site is approximately 80 km north of the city Manaus (Amazonas, Brazil).

Leaves of three specimens of *Ocotea delicata* were sampled within a perimeter of 80 m. The leaves of each specimen were treated as an individual sample, totaling three samples. These specimens are constantly monitored by PDBFF under the identification numbers 50573, 40608, and 74168. Vouchers of each specimen were labeled and deposited in the Herbarium of the Federal Institute of Education, Science and Technology of Amazonas (EAFM) in Manaus, Amazonas, under deposit numbers 16640, 16642, and 16680, corresponding to samples A, B, and C, respectively. The location of the specimens at the sample site was as follows: A = 02°26.613′ S, 059°47.245′ W (50573); B = 02°26.616′ S, 059°47.249′ W (40608); and C = 02°26.506′ S, 059°47.246′ W (74168). The botanical identification of specimens was performed by Dr. Alberto Vicentini (INPA). The specimens were registered in the National System of Genetic Heritage and Associated Traditional Knowledge (SISGEN) under the number A02836A.

### 4.2. Sampling Preparation

The leaves of each sample, collected from a single branch, were cleaned, air-dried, and grounded to a particle size of 0.595 mm with a knife mill (Wiley Mill SP-32, SPLABOR, São Paulo, Brazil). Two grams of each of the dried samples were subjected to ultrasound-assisted extraction in an ultrasonic bath (Ultrasonic Cleaner, Unique, São Paulo, Brazil) operating at 40 kHz for 30 min at 40 °C. A hydroalcoholic solution of 70% ethanol was used as an extracting solvent in a proportion of 1:12 (g:mL) between sample and extractor solvent volume. The ethanolic extracts were evaporated until dry through distillation in a rotary evaporator (Fisatom 802, São Paulo, Brazil) under reduced pressure. For each sample, three extracts were produced in order to generate triplicates from the same branch. The extraction method selection was based on previous metabolomics studies within the *Ocotea* genus [21].

The crude extracts obtained were individually weighed (2 mg), diluted in an hydroalcoholic solution of 80% methanol, degreased with hexane, and partitioned with ethyl acetate. The ethyl acetate fraction was dried using rotary evaporation. Chemical analysis was conducted using the ethyl acetate fraction, as the available *Ocotea* and Lauraceae literature indicates that the medium polarity fraction contains several of its chemical markers and bioactive compounds [3,7,22,23].

### 4.3. Chemical Profile Analysis

The chemical profiles were obtained using Ultra High-Performance Liquid Chromatography coupled with High-Resolution Mass Spectrometry (UHPLC-HRMS). Before analysis, each sample was prepared at 1 mg mL^−1^ with methanol and filtered using 0.45 µm Polytetrafluoroethylene (PTFE) with Glass Micro Fiber (GMF) membranes (Whatman, Little Chalfont, UK). UHPLC-MS analyses were performed using a Dionex Ultimate 3000 UHPLC (Thermo Scientific, Bremen, Germany) connected to a Q-Exactive high resolution spectrometer (Thermo Scientific, Bremen, Germany). Chromatographic analysis was carried out with a Syncronis C18 (2.1 × 50 mm, 100 Å—Thermofisher Scientific, Waltham, MA, USA). Mobile phase was composed of two solvents, Solvent A and B. Solvent A was a solution of 0.1% formic acid in deionized water/ammonium formiate 5 mM and Solvent B was methanol 100%. Elution was performed in a gradient mode from 0 to 100% of Solvent B from 0 to 8 min, followed by 100% of Solvent B for 1 min. Mobile phase flow was set at 0.400 mL min^−1^. Samples were injected at a volume of 8.0 µL with an injection system and oven temperature of 40 °C. The spectrometer used an electrospray ionization source operating in positive and negative ionization modes at a range of 100 to 900 *m*/*z* in a data-dependent acquisition mode for MS^2^ fragmentation experiments. Mass spectrometry data were obtained with sheath and auxiliary gas flow rate at 60 and 20 arb, respectively; a spray voltage of 3.9 kV (positive mode) and 2.9 kV (negative mode); capillary temperature at 380 °C; a resolution of 70,000 FHWM; an isolation window of *m*/*z* 4.0 range; an collision energy of 30 eV; and a mass error of 5.0 ppm. Each sample (n = 3) was analyzed in triplicate.

### 4.4. Data Processing

Data processing was performed in the MZMine 2.53v software. Peak detection was performed through a workflow containing the following steps: baseline correction filtering; mass detection of *m*/*z* signals with the exact mass algorithm; chromatogram building with the ADAP algorithm; chromatogram deconvolution with the baseline cut-off algorithm; and isotope identification; and it was aligned with the Join Aligner algorithm [24,25,26]. Supplementary Table 1 list the parameter values used in each step. The suggestive identification of detected *m*/*z* signals was performed through a spectral library search within the Global Natural Products Social Molecular Networking (GNPS) [24,27,28,29]. Putative identification on GNPS was based on a similarity score among experimental MS^2^ fragmentation spectra and library with at least three identical fragments peaks and a cosine score larger than 0.7 (in a scale ranging from 0 (no similarity) to 1 (identical)) [30].

Descriptive and multivariate statistics were performed using the software OriginPro 2017. The detected *m*/*z* signals were evaluated through Hierarchical Clustering Analysis (HCA) with Ward’s method and Euclidian distance, and Principal Component Analysis (PCA) to evaluate sample similarity patterns.

## 5. Conclusions

This study represents the first phytochemistry analysis of *Ocotea delicata*, enabling the identification of 44 metabolites. The profiles of flavonoids and alkaloids within the samples were found to correlate with the biomass of tree specimens. *O. delicata* exhibited chemical features similar to those of other New World *Ocotea* species, with the simultaneous occurrence of flavonols, aporphine, and benzylisoquinoline alkaloids. The relative concentration of benzylisoquinoline alkaloids could serve as a chemical marker for studies on intraspecific variability in *Ocotea*. Further research employing a larger sample set of *Ocotea* species is necessary to confirm the accuracy of these chemical markers for intraspecific variability studies, validate the chemical profile of *O. delicata*, and discover chemical markers capable of distinguishing between different species.

## Figures and Tables

**Table 1:** Putative compounds identified within *O. delicata* samples (A, B, and C) using UHPLC-HRMS and GNPS, and specimen (A, B, and C) features.

					Relative Peak Area					
					Specimen A		Specimen B		Specimen C	
Number	Metabolites	RT	Detected *m*/*z*	*m*/*z* Error (ppm)	Mean (n = 3)	±RSD	Mean (n = 3)	±RSD	Mean (n = 3)	±RSD
	Negative Ionization Mode									
1	Raffinose	0.33	503.162	0.788	0.89 ^a^	0.01	1.04 ^a^	0.10	1.14 ^a^	0.06
2	Quinic acid	0.33	191.055	4.712	26.38 ^a^	0.38	58.56 ^b^	6.82	19.25 ^c^	3.50
3	5-Feruloylquinic acid	3.83	367.103	0.831	0.29 ^a^	0.02	0.08 ^a^	0.07	8.18 ^b^	0.89
4	4-O-Caffeoylquinic acid	3.84	353.088	0.864	n.d		n.d		22.49	3.28
5	Boldine	3.94	326.140	0.936	11.41 ^a^	19.73 ^a^	30.88	5.48	n.d	0.00
6	Cianidanol	4.11	289.072	1.372	n.d	0.00	0.02 ^a^	0.01	0.06 ^a^	0.01
7	2-[3,4-dihydroxy-4-(hydroxymethyl)oxolan-2-yl]oxymethyl]-6-(2-phenylethoxy)oxane-3,4,5-triol	4.55	415.161	0.221	5.53 ^a^	9.55	5.64 ^a^	3.00	n.d	
8	Euriocitrin	4.69	593.151	0.514	1.93 ^a^	0.08	1.49 ^a^	0.04	n.d	
9	Calceolarioside A	4.97	477.140	0.256	0.01 ^a^	0.01	n.d		0.11 ^b^	0.04
10	Quercetin 3,7-dirhamnoside	5.21	593.151	0.206	n.d		0.04	0.03	n.d	
11	Hyperoside	5.25	463.088	0.857	n.d		n.d		0.88	0.19
12	Kaempferol 7-O-glucoside	5.33	447.093	0.956	n.d		0.12 ^a^	0.03	0.01 ^b^	0.02
13	Avicularin	5.34	433.078	0.916	n.d		n.d		0.08	0.03
14	Azelate	5.54	187.097	7.014	1.13 ^a^	0.11	1.53 ^a^	0.06	0.10 ^b^	0.01
15	Nicotiflorin	5.55	593.151	0.514	0.10 ^a^	0.01	1.74 ^b^	0.12	0.02 ^a^	0.00
16	3-phenyl-2-3.4.5-trihydroxy-6-[3-(4-hydroxyphenyl)prop-2-enoyl]oxymethyl]oxan-2-yl]oxyprop-2-enoic acid	5.56	471.130	0.842	n.d		0.76 ^a^	0.12	0.04 ^b^	0.00
17	Quercitrin	5.56	447.093	0.683	0.19 ^a^	0.03	5.30 ^b^	0.83	0.52 ^a^	0.17
18	Abscisic acid	5.70	263.129	0.696	0.26 ^a^	0.03	0.39 ^b^	0.09	0.01 ^c^	0.01
19	Kaempferol 3-arabinoside	5.71	417.083	0.293	0.05 ^a^	0.04	0.18 ^b^	0.03	n.d	
20	3″,4″-Di-O-p-coumaroylafzelin	7.88	723.173	0.591	0.01 ^a^	0.01	0.08 ^a^	0.03	0.01 ^a^	0.01
21	Afzelin	9.19	174.955	0.920	1.69 ^a^	1.46	n.d		0.86 ^a^	0.80
	Positive ionization mode									
22	Salsolinol	0.33	180.102	1.101	0.04 ^a^	0.00	0.23 ^b^	0.02	15.60 ^c^	2.82
4	4-O-Caffeoylquinic acid	3.01	377.084	0.486	n.d		n.d		0.12	0.13
23	N-methylcoclaurine N-oxide	3.65	316.154	2.799	0.02 ^a^	0.02	0.02 ^a^	0.04	n.d	
24	Feruloyltyramine	3.70	265.155	5.294	n.d		n.d		0.07	0.07
25	Chlorogenic acid	3.77	377.084	0.000	n.d		n.d		0.78	0.44
26	Ferulate	3.81	177.055	2.844	n.d		n.d		0.18	0.02
27	Syringin	3.91	395.131	0.463	0.01 ^a^	0.01	0.01 ^a^	0.00	0.06 ^a^	0.04
28	Coclaurine	3.92	286.144	1.493	0.17 ^a^	0.04	8.50 ^b^	0.38	0.33 ^a^	0.57
29	Fraxetin	4.03	209.044	1.898	n.d		n.d		0.12	0.04
30	Fraxin	4.03	393.079	0.000	n.d		n.d		0.49	0.30
31	Procyanidin B2	4.05	579.149	1.054	n.d		n.d		0.04	0.01
32	Reticuline	4.08	330.170	0.924	38.40 ^a^	2.49	22.09 ^b^	0.43	0.22 ^c^	0.06
5	Boldine	4.15	328.154	0.372	8.59 ^a^	9.14	38.14 ^a^	8.89	0.02 ^b^	0.01
33	Epicatechin	4.16	291.086	3.145	n.d		n.d		0.02	0.01
34	Isocorydine	4.28	342.170	0.624	23.35 ^a^	1.25	1.94 ^b^	3.35	0.10 ^b^	0.02
35	Reticuline N-oxide	4.32	346.165	0.882	1.52 ^a^	0.12	0.03 ^b^	0.04	0.09 ^b^	0.01
36	N-methylcoclaurine	4.45	300.159	2.338	0.04 ^a^	0.00	0.12 ^a^	0.10	n.d	
37	Isofraxidin	4.76	223.060	0.000	n.d		n.d		0.03	0.02
38	Taxifolin	5.06	305.065	1.300	n.d		n.d		0.02	0.01
39	3.5-Dicaffeoylquinic acid	5.06	499.123	0.428	n.d		n.d		0.005	0.001
40	Rutin	5.25	633.142	1.446	n.d		n.d		0.05	0.01
41	Phlorhizin	5.32	459.126	0.665	n.d		n.d		0.003	0.002
42	Datiscetin	5.93	287.055	0.319	0.02 ^a^	0.01	0.26 ^b^	0.07	0.03 ^a^	0.03
43	3.7-Dimethyl-2.6-octadien-1-yl 6-O-[3.4-dihydroxy-4-(hydroxymethyl)tetrahydro-2-furanyl]-β-D-glucopyranoside	6.28	471.220	0.453	n.d		0.02 ^a^	0.00	0.21 ^b^	0.11
44	N-methylaurotetanine	7.79	342.170	0.000	0.01	0.00	n.d		n.d	
	Additional features regarding specimens									
	Specimen				A	B	C			
	Diameter at breast height (mm)				29	40	20			
	Relief elevation (m)				124	120	120			

RT = retention time (min); n = number of replicates evaluated from each specimen; RSD = relative standard deviation; n.d = not detected; different upper letters (^a, b^ and ^c^) represent statistically different means between specimens.

## Data Availability

Data are contained within the article or supplementary material.

## Supplementary Materials

The following supporting information can be downloaded at: https://www.mdpi.com/article/10.3390/plants13060859/s1. Table S1. Parameters applied during chromatographic data curation within MZmine 2.53v and the GNPS platform; Table S2. *m*/*z* signals relevant to sample distinction using Principal Component Analysis; Figure S1. Putatively identified metabolites on negative ionization mode (metabolite identification in accordance with Table 1); Figure S2. Putatively identified metabolites on positive ionization mode (metabolite identification in accordance with Table 1); Figure S3. Extracted-ion chromatogram of *O. delicata* samples in negative ionization mode; Figure S4. Extracted-ion chromatogram of *O. delicata* samples in positive ionization mode.
