# Supplementary material for: Chemical Profile of Ocotea delicata (Lauraceae) Using Ultra High-Performance Liquid Chromatography–High-Resolution Mass Spectrometry–Global Natural Products Social Molecular Networking Workflow
Source: Plants (Basel). 2024 Mar 16;13(6):859. doi: 10.3390/plants13060859 (PMC10975221; doi:10.3390/plants13060859)

SUPPLEMENTARY MATERIAL

Chemical profile of *Ocotea delicata* (Lauraceae) using Ultra High-Performance Liquid Chromatography–High-Resolution Mass Spectrometry–Global Natural Products Social Molecular Networking workflow

Ananda da Silva Antonio [1,2], Gustavo Ramalho Cardoso dos Santos [1], Henrique Marcelo Gualberto Pereira [1], Valdir Florêncio da Veiga-Juniora [3,*], Larissa Silveira Moreira Wiedemann [1]

[1] Department of Chemistry, Institute of Exact Sciences, Federal University of Amazonas, Avenida Rodrigo Otávio, 6200, Coroado, 69077-000, Manaus/AM-Brazil;

[2] Chemistry Institute, Federal University of Rio de Janeiro, Avenida Horácio Macedo, 1281 - Polo de Química - Cidade Universitária, Ilha do Fundão, CEP: 21941-598, Rio de Janeiro/RJ-Brazil;

[3] Department of Chemical Engineering, Military Institute of Engineering – IME, Praça General Tiburcio 80, Urca, 22290-270, Rio de Janeiro/RJ – Brazil;

* Correspondence: valdir.veiga@gmail.com

Abstract: *Ocotea*, the largest genus in the Lauraceae family, encompasses numerous species of scientific interest. However, most *Ocotea* species have only been described morphologically. This study used an untargeted metabolomics workflow with UHPLC-HRMS and GNPS-FBMN to provide the first chemical evaluation of the polar specialized metabolites of *O. delicata* leaves. Leaves from three *O. delicata* specimens were extracted using ultrasound-assisted extraction with 70% ethanol. Among the examined samples, 44 metabolites, including alkaloids and flavonoids, were identified. In contrast to other *Ocotea* species, *O. delicata* has a wider diversity of kaempferol derivatives than quercetin. The biomass of the specimens showed a significant correlation with the chemical profile. The similarity among specimens was mostly determined by the concentrations of quinic acid, kaempferol glycosides, and boldine. The evaluated specimens exhibited chemical features similar to those of species classified as New World *Ocotea*, with the coexistence of aporphine and benzylisoquinoline alkaloids.

Keywords: untargeted metabolomics; chemosystematic markers; chemophenetics

Table S1. Parameters applied during chromatographic data curation within MZmine 2.53v and GNPS platform

Baseline correction	
Chromatogram type	TIC
MS level	1
M/z bin width	1
Correction method	Asymmetric baseline corrector
Smoothing	1.00E+05
Asymmetry	0.05
Mass detection	
Mass detector	Exact mass
MS level	1
Noise level	1.50E+04
MS level	2
Noise level	2.00E+03
ADAP chromatogram builder	
Min group size of scan	5
Group intesity threshold	1.50E+04
Min highest intensity	1.50E+04
M/z tolerance \(ppm)	10
Chromatogram deconvolution	
Algorithm	Baseline cut-off
Min. Peak height	1.50E+04
Peak duration (min)	0 to 3
Baseline level	4.50E+04
M/z range for MS2 scan pairing (Da)	0.01
M/z range for MS2 scan pairing (min)	0.2
M/z center calculation	Average
Deisotope	
M/z tolerance \(ppm)	10
Retention time tolerance (%)	0.1
Maximum charge	3
Representative isotope	Most intense
Join aligner	
M/z tolerance \(ppm)	10
Weight for m/z	75
Retention time tolerance min.	0.1
Weight for RT	25
Filtering	
Min. Peak in a row	2
Min. Peak in na isotope pattern	2
Keep only with MS2 scan (GNPS)	Yes
GNPS-FNMB	
Precursor ion mass tolerante (Da)	2
Fragment ion mass tolerance (Da)	0.5

Table S2. *m/z* signals relevant to samples distinction using Principal Components Analysis.

Negative ionization mode							
		Relative peak area					
		A		B		C	
Detected *m/z*	Retention time (min.)	Mean	± SD	Mean	± SD	Mean	± SD
879.177	3.52	0.00	0.00	0.00	0.00	100.00	0.00
863.183	3.78	0.00	0.00	0.00	0.00	76.72	1.79
165.039	0.33	46.07	1.92	99.46	0.93	7.46	1.63
723.172	7.51	15.52	1.78	58.18	2.63	1.63	0.28
179.055	0.33	100.00	0.00	69.19	4.67	1.19	0.22
191.055	3.02	0.00	0.00	0.00	0.00	0.32	0.06
431.098	5.98	0.36	0.32	33.33	57.74	0.01	0.00
374.109	3.64	61.19	12.87	16.17	3.65	0.00	0.00
312.124	3.91	2.48	4.27	68.87	15.00	0.00	0.00
**Positive ionization mode**							
		Relative peak area					
		A		B		C	
Detected *m/z*	Retention time (min.)	Mean	± SD	Mean	± SD	Mean	± SD
274.274	7.061	34.15	3.38	18.50	2.62	100.00	0.00
280.109	4.564	52.19	1.69	57.75	1.12	0.00	0.00
295.096	4.682	100.00	0.00	76.97	0.49	0.00	0.00
311.128	4.552	91.71	2.25	98.47	1.73	0.00	0.00
312.123	4.683	79.13	4.23	61.12	2.72	0.00	0.00
322.071	5.801	61.83	2.58	39.01	1.07	0.00	0.00
328.154	4.564	80.31	3.12	82.46	0.77	0.00	0.00
328.154	4.157	8.59	9.14	38.14	8.89	0.02	0.01
330.170	4.084	38.40	2.49	22.09	0.43	0.22	0.05
338.102	6.247	55.84	3.55	47.27	1.53	0.00	0.00
342.170	4.513	79.20	4.51	97.95	3.55	0.01	0.00

SD = standard deviation

Figure S1. Putatively identified metabolites on negative ionization mode (metabolites identification in accordance with Table 1).

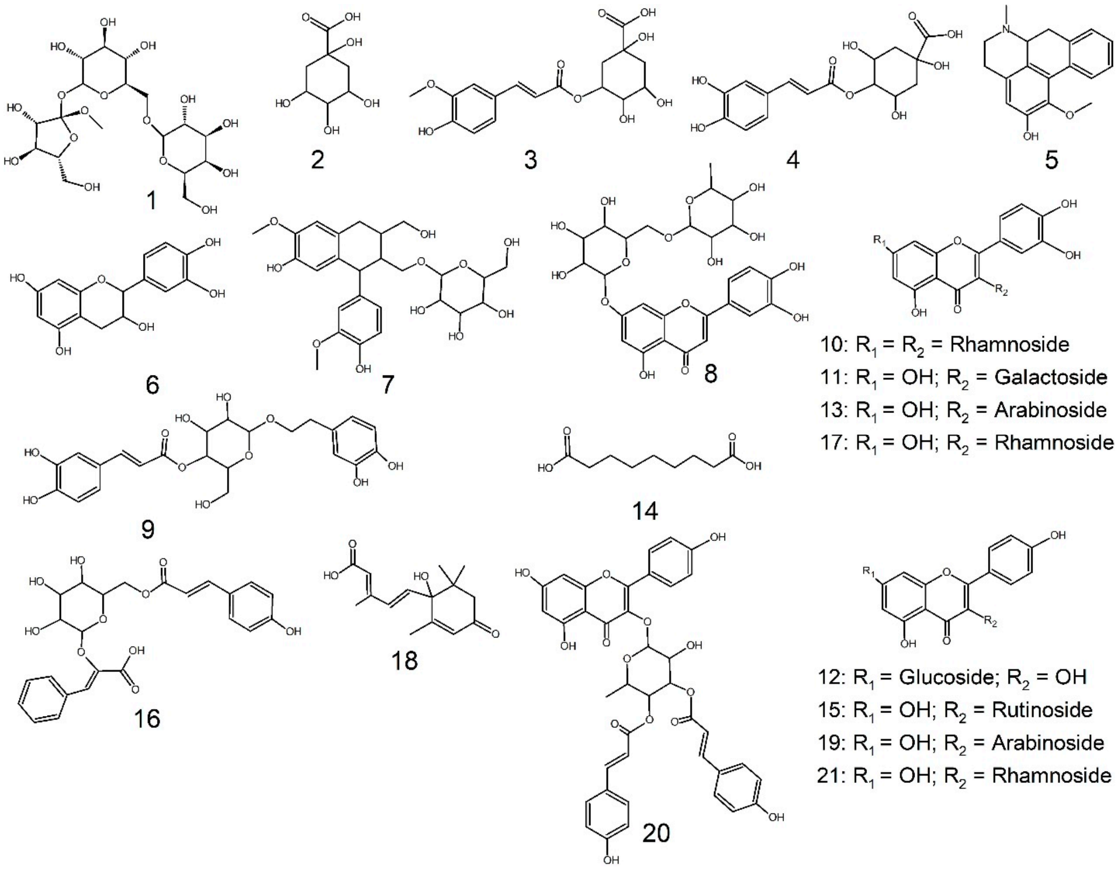

Figure S2. Putatively identified metabolites on positive ionization mode (metabolites identification in accordance with Table 1).

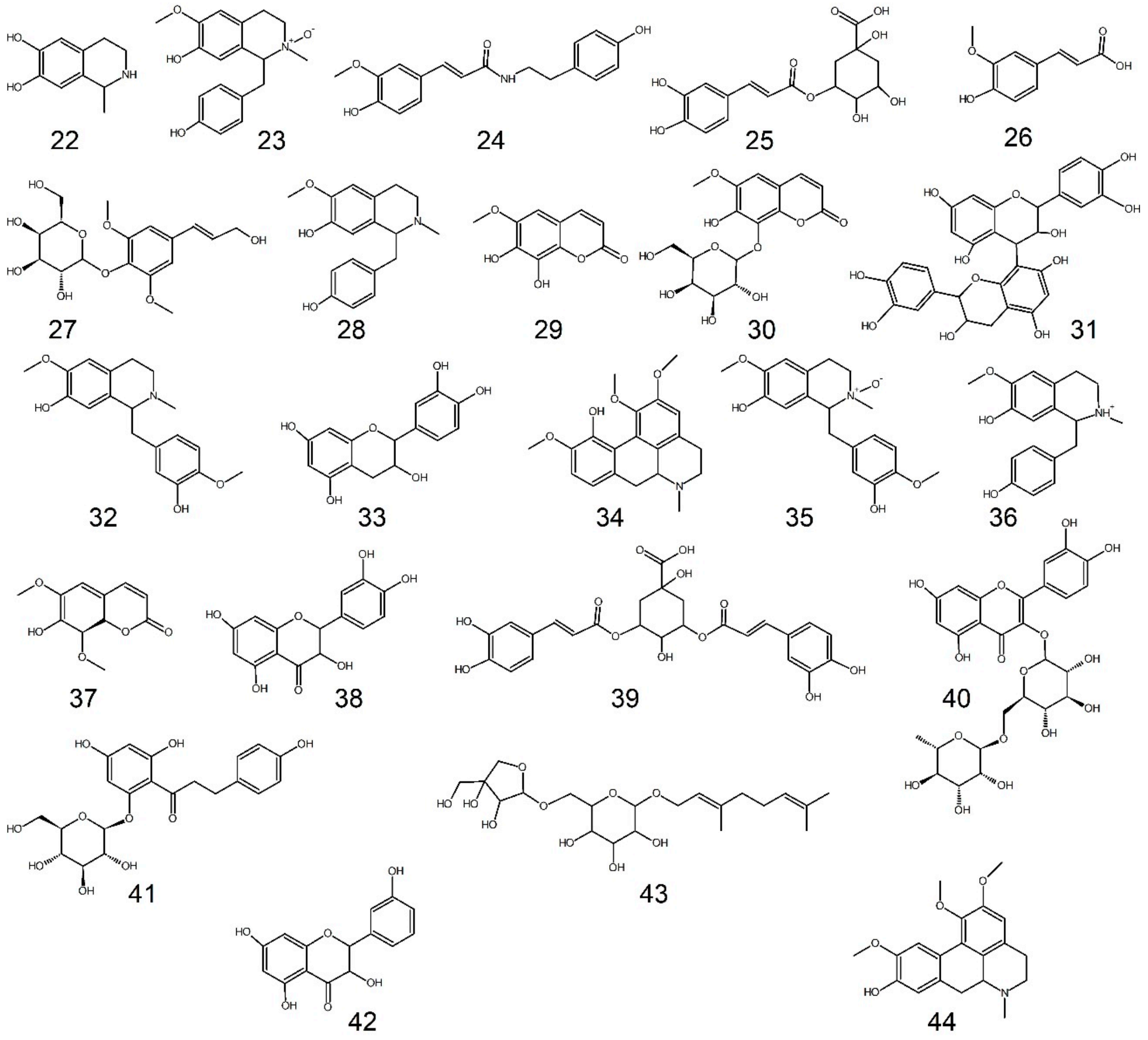

Figure S3. Extracted-ion chromatogram of *O. delicata* samples in negative ionization mode.

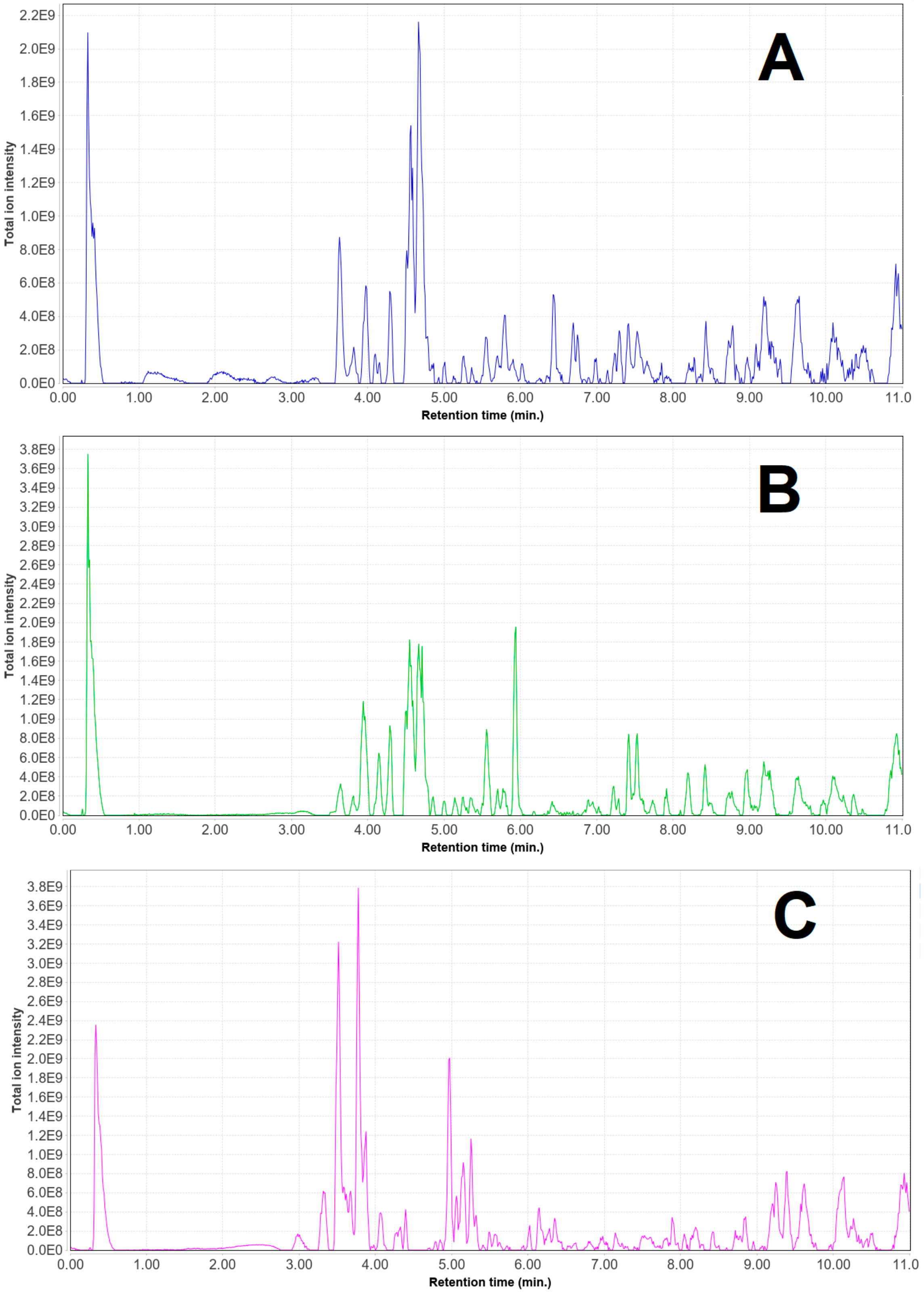

Figure S4. Extracted-ion chromatogram of *O. delicata* samples in positive ionization mode.

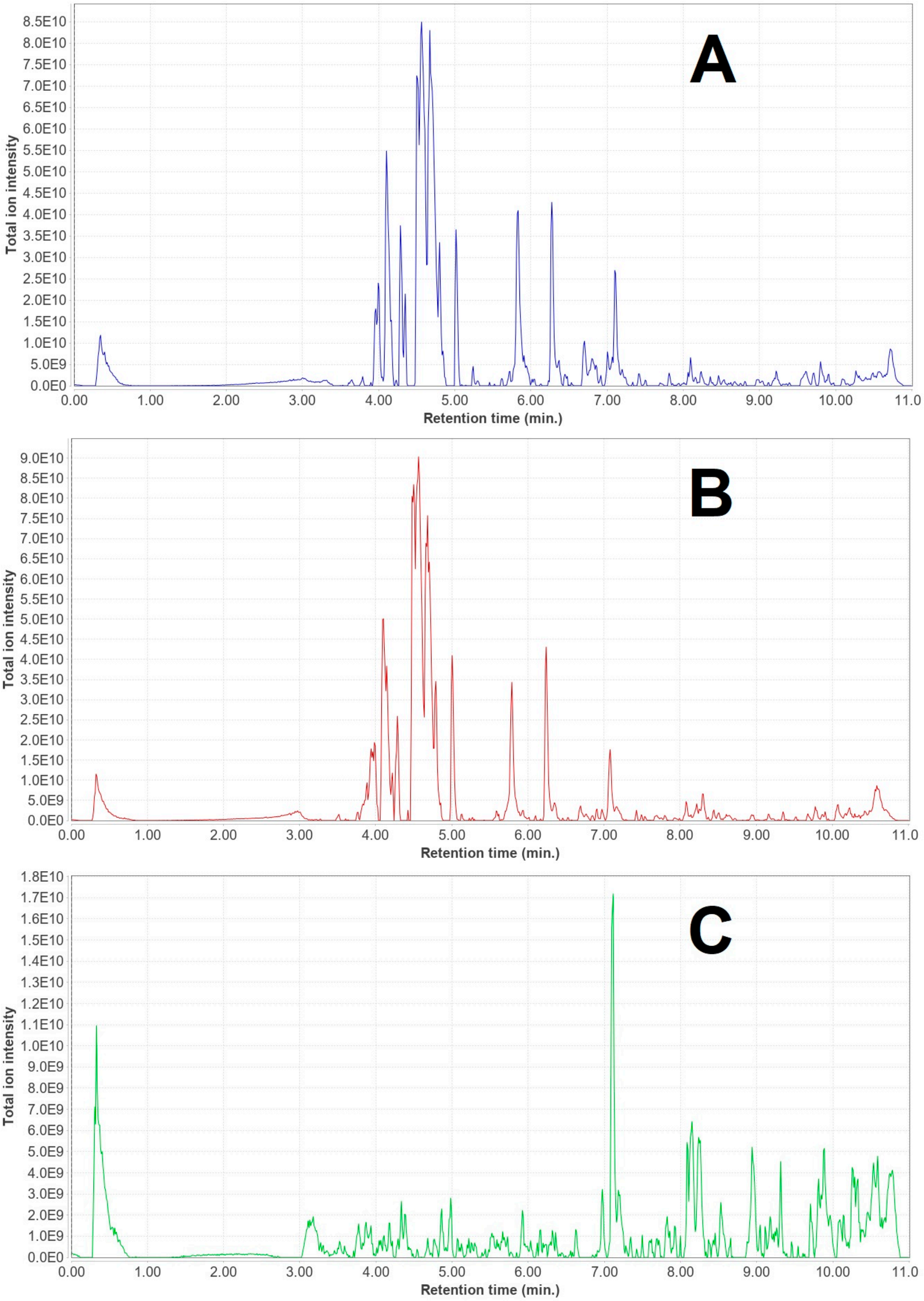

Supplement: Supplementary file 1 [file plants-13-00859-s001.zip › plants-2895292-supplementary.pdf]
